# Analysis of transcription factors among differentially expressed genes induced by drought stress in *Populus davidiana*

**DOI:** 10.1007/s13205-017-0858-7

**Published:** 2017-06-30

**Authors:** Bong-Gyu Mun, Sang-Uk Lee, Eung-Jun Park, Hyun-Ho Kim, Adil Hussain, Qari Muhammad Imran, In-Jung Lee, Byung-Wook Yun

**Affiliations:** 1 School of Applied Biosciences, Kyungpook National University, Daegu, 41566 Republic of Korea; 2 Division of Forest Biotechnology, Korea Forest Research Institute, Suwon, 16631 Republic of Korea; 3 Department of Agriculture, Abdul Wali Khan University, Mardan, Pakistan

**Keywords:** Drought stress, Transcriptome, Poplar, Transcription factors

## Abstract

*Populus davidiana* is native to the Korean Peninsula and is one of the most dominant and abundantly growing forest trees in eastern Asia. Compared to other *Populus* species such as *P. trichocarpa, P. euphratica,* and *P. tremula*, relatively little is known about *P. davidiana.* Here, we performed transcriptomic analysis of *P. davidiana* under drought stress induced by 10% polyethylene glycol. A total of 12,403 and 12,414 differentially expressed genes (DEGs) were successfully annotated with the *P. trichocarpa* reference genome after 6 and 12 h of treatment, respectively. Of these, a total of 404 genes (238 up-regulated and 166 down-regulated) after 6 h and 359 genes (187 up-regulated and 172 down-regulated) after 12 h of treatment were identified as transcription factors. Transcription factors known to be key genes for drought stress response, such as AP2-EREB, WRKY, C2H2, and NAC, were identified. This results suggesting that early induction of these genes affected initiation of transcriptional regulation in response to drought stress. Quantitative real-time PCR results of selected genes showed highly significant (*R* = 0.93) correlation with RNA-Seq data. Interestingly, the expression pattern of some transcription factors was *P. davidiana* specific. The sequence of *P. davidiana* ortholog of *P. trichocarpa* gene POPTR_0018s10230, which plays an important role in plant response to drought, was further analyzed as our RNA-Seq results showed highly significant changes in the expression of this gene following the stress treatment. Sequence of the gene was compared to *P. trichocarpa* gene sequence using cloning-based sequencing. Additionally, we generated a predicted 3D protein structure for the gene product. Results indicated that the amino acid sequence of *P. davidiana*-specific POPTR_0018s10230 is different at six different positions compared to *P. trichocarpa*, resulting in a significantly different structure of the protein. Identifying the transcription factors expressed in *P. davidiana* under drought stress will not only offer clues for understanding the underlying mechanisms involved in drought stress physiology but also serve as a basis for future molecular studies on this species.

**Electronic supplementary material:**

The online version of this article (doi:10.1007/s13205-017-0858-7) contains supplementary material, which is available to authorized users.

## Introduction

*Populus davidiana* is a plant species native to the Korean Peninsula and is one of the most widely distributed forest trees in Korea. The ubiquity of *Populus* species is indicative of their ability to adapt to diverse environmental conditions, such as cold (Chen et al. 2012), salt (Gu et al. 2004), and drought (Li et al. 2011b; Tang et al. 2013b). Drought occurs when there is insufficient irrigation or rainfall for a period, such that soil moisture is reduced to an extent that ultimately damages or injures plants. This deficiency is typically accompanied by higher evapotranspiration rates from plant surfaces compared to water absorption by the roots (Jordan and Ritchie 1971).

Drought stress has also been found to be accompanied by other abiotic stresses such as salinity and high temperature stress. Salt and drought stress signal transduction consists of ionic and osmotic homeostasis, detoxification, and growth regulation. The adverse effects of water stress on plant physiology and the mechanisms associated with water stress tolerance and water-use efficiency have been extensively studied (Osakabe et al. 2014). Although *Populus* trees have a much deeper root system compared to agricultural corps, they are still affected by persistent drought. Persistent drought can influence the structure and growth of roots which in turn negatively affects water uptake leading to the appearance of initial drought symptoms and permanent damage if drought persists (Coder 1999; Kozlowski and Pallardy 2002). Published researches on the molecular mechanisms underpinning responses to drought stress in various crops, such as maize (Avramova et al. 2014), barley (Bedada et al. 2014), potato (Gong et al. 2015), rice (Huang et al. 2014), wheat (Okay et al. 2014), sugarcane (Kido et al. 2012), and soybean (Le et al. 2012), and many other plants, including forest trees such as poplar, pine, and oak (Dong et al. 2014a; Li et al. 2011a). These studies provide useful information regarding the underlying mechanisms and possible management of the problem.

*Populus* is a promising model of forest trees and/or other woody plants for research on diverse stress responses (Li et al. 2011a; Qiu et al. 2011; Si et al. 2014; Yan et al. 2012). Moderately drought-stressed *Populus euphratica* trees have been found to regulate stomatal closure to facilitate higher CO_2_ accumulation and water absorption for normal growth and development. This is typically accompanied by strong transcriptional regulation of various physiological processes such as stress perception, photoreception, and oxidative stress detoxification at the molecular level (Tang et al. 2013a). Several studies have shown that some species of *Populus*, such as *P. euphratica,* are extremely sensitive to drought-induced cavitation (Hukin et al. 2005), whereas *P. nigra* shows tolerance to drought. Plant cellular responses to various biotic and/or abiotic stresses involve highly complex interconnected networks of signaling pathways, and a systematic understanding of these networks is necessary to comprehend the underlying mechanisms of stress tolerance. An efficient approach for examining the complex internal networks initiated in response to drought stress is discovering genes and metabolic pathways involved in drought stress physiology. This approach may provide clues for the production of drought-tolerant plants (Hamanishi and Campbell 2011).

Recently, high-throughput sequencing technologies have yielded accurate whole-genome sequences on a large scale at low-cost and in a relatively short time. To date, RNA-seq-mediated transcriptome analysis of three *Populus* species (*P. tremula* (Paul et al. 2014), *P. euphratica* (Chen et al. 2014), and *P. trichocarpa* (Kumar et al. 2016) has been reported. More recently, five genes involved in the tolerance to salts, drought, waterlogging, and insect attack have been identified from a transgenic poplar line (*Populus* × *euramericana* ‘Guariento’) based on transcriptome analysis (Zhang et al. 2014).

Transcription factors (TFs) are proteins that regulate the transcription of gene expression by binding to a certain sequence of DNA or/and other protein complex, thereby altering the activity of a protein by either promoting or suppressing its function. Several studies have reported the important role of TFs in response to biotic and abiotic stresses in *Populus* (Wang et al. 2017). Examples include the NAC and WAKY TFs, as large gene families, involved in plant growth regulation, developmental processes, and stress responses. Although information on the TFs in several *Populus* species, including *P. trichocarpa* (Hu et al. 2010) and *P. euphratica* (Ma et al. 2016), has been reported, little information is available on the TFs in *P. davidiana*, particularly under drought stress conditions.

Here, we present a transcriptomic analysis of *P. davidiana* and, in particular, the TF families induced by polyethylene glycol (PEG) treatment. PEG is used for induction of high osmotic pressure, thereby causing an effect in vitro similar to drought stress (Muscolo et al. 2014). We anticipate that the findings of this study will serve as a basis for future research on drought stress physiology in *Populus* species.

## Materials and methods

### Plant growth and stress treatment

*P. davidiana* seeds were surface-sterilized in 3% H_2_O_2_, rinsed several times with sterile water (Lendzemo et al. 2009), and then germinated on half-strength Murashige and Skoog (MS) medium (MS 2.2 g, sucrose 10 g, plant agar 8 g; adjusted to pH 5.8). Four-week-old plants were explanted on MS [4.4 g MS, 3% sucrose, 0.27% Gelrite, final pH 5.8; supplemented with 0.5 ppm naphthalene acetic acid (NAA)]. The plants were then incubated for 4 weeks in a controlled environment (22–23 °C, 16 h light and 8 h dark) for organogenesis. Eight-week-old plants were used for stress treatment. Drought stresses were induced by treatment with 10% polyethylene glycol (PEG) as described by Kwon et al. (2014). All the treatments were performed in triplicates, and samples were collected from analysis at 6 and 12 h after PEG treatment.

### RNA extraction and sequencing

Total RNA was extracted using Trizol (Invitrogen). Briefly, *P. davidiana* leaf tissues were finely ground in liquid nitrogen using a pestle and mortar and immediately homogenized in 1 ml of Trizol. The homogenized samples were centrifuged (13,000*g*, 10 min, 4 °C), and the supernatant was transferred to fresh tubes. After adding 200 µl chloroform, the tubes were vortexed vigorously, kept on ice for 3 min, and then centrifuged. The upper aqueous phase was transferred to fresh tubes containing an equal volume of isopropanol. These tubes were then centrifuged at 4 °C. Precipitated RNA was washed with 75% ethyl alcohol and re-suspended in RNase-free water.

Sequencing was carried out as described (Hussain et al. 2016). Briefly, RNA libraries were generated using a TruSeq RNA library prep kit v2 (Illumina, USA) and then cDNA was synthesized through fragmentation and hexamer priming of mRNA. Generated cDNA libraries were quantified using a KAPA library quantification kit. A Hiseq-2500 sequencer (Illumina, USA) was used for the RNA-seq.

### Measurement of expression levels and identification of differentially expressed genes

High-quality reads from the raw sequencing reads were matched to the *P. trichocarpa* genome using Ensembl (v.26) (Flicek et al. 2014), and the TopHat program was applied for alignment of RNA-seq reads. Expression levels of genes in the transcriptome were calculated using Cufflinks v2.2.1 (Trapnell et al. 2010) and compared to the *P. trichocarpa* reference data. Additionally, to increase the accuracy of the gene expression level measurements, the data were subjected to multi-read correction and frag-bias corrections. Thereafter, DEGs were identified based on FPKM (fragments per kilo base of transcript per million mapped fragment) and *Q* value (<0.05) (Q value: error-corrected value after multiple testing). Further, to verify the statistical significance and hierarchical clustering of DEGs, a Heat map was generated using *R* software (version 3.3.1).

### Functional categorization of TFs and comparative analysis

*P. davidiana* genes were further annotated and analyzed with respect to gene ontology (GO) terms. For this, we referred to the following online databases: NCBI (www.ncbi.nlm.nih.gov), PopGenie (https://popgenie.org), DPTF (https://dptf.cbi.pku.edu.cn), and Plant TFDB (planttfdb_v2.cbi.edu.cn). Similarity BLAST searches were conducted using the NCBI database. GO enrichment analysis was conducted using GO-slim and classified to molecular function, biological process, and cellular location. Transcription factors were analyzed using MapMan (version 3.6.0) to classify functional groups based on a comparison with *P. trichocarpa* as a reference genome from PopGenie and Phytozome (https://phytozome.jgi.doe.gov/). The expression levels of DEGs were validated by qRT-PCR from 10 selected genes, and SNP/indel positions in three genes were compared between *P. davidiana* and *P. trichocarpa* and identified using the Integrative Genomics Viewer (IGV 2.3.72) program.

### Structural prediction and protein modeling

Comparison of the *P. davidiana* sequence with that of *P. trichocarpa* was carried out using the IGV program. The amino acid sequences of selected transcription factors of the two poplar species were submitted to the I-TASSER server for protein model prediction. I-TASSER produced one to five models for each of the poplar sequences. Among these, a model with the highest confidence score (C-score) was selected and analyzed using PyMOL software (LLC, http://www.pymol.org/).

### Transcriptomic data analysis and statistical analysis

To obtain high-quality reads from the raw reads, low-quality reads and adaptors were discarded as described by (Hussain et al. 2016). Briefly, the reads with >10% ambiguous bases or with *Q* 20 < 40% were removed and then quality control was performed using the in-house Theragen software (Theragen Etex, Suwon, Korea). High-quality reads were aligned using TopHat (Trapnell et al. 2009). Expression levels were measured using Cufflinks v2.2.1 (Trapnell et al. 2010). After frag-bias correction and multi-read corrections, differences in the expression of genes were calculated through Cufdiff v2.2.1 (Trapnell et al. 2010) to identify differentially expressed genes with *Q* < 0.05. To check correlation between qRT-PCR and RNA-Seq analysis, the log-fold change values of transcripts from both the experiments were plotted in Microsoft Excel 2007 and statistically analyzed to calculate the correlation coefficient *R*.

## Results

### Transcriptome analysis

**Fig. 1:**
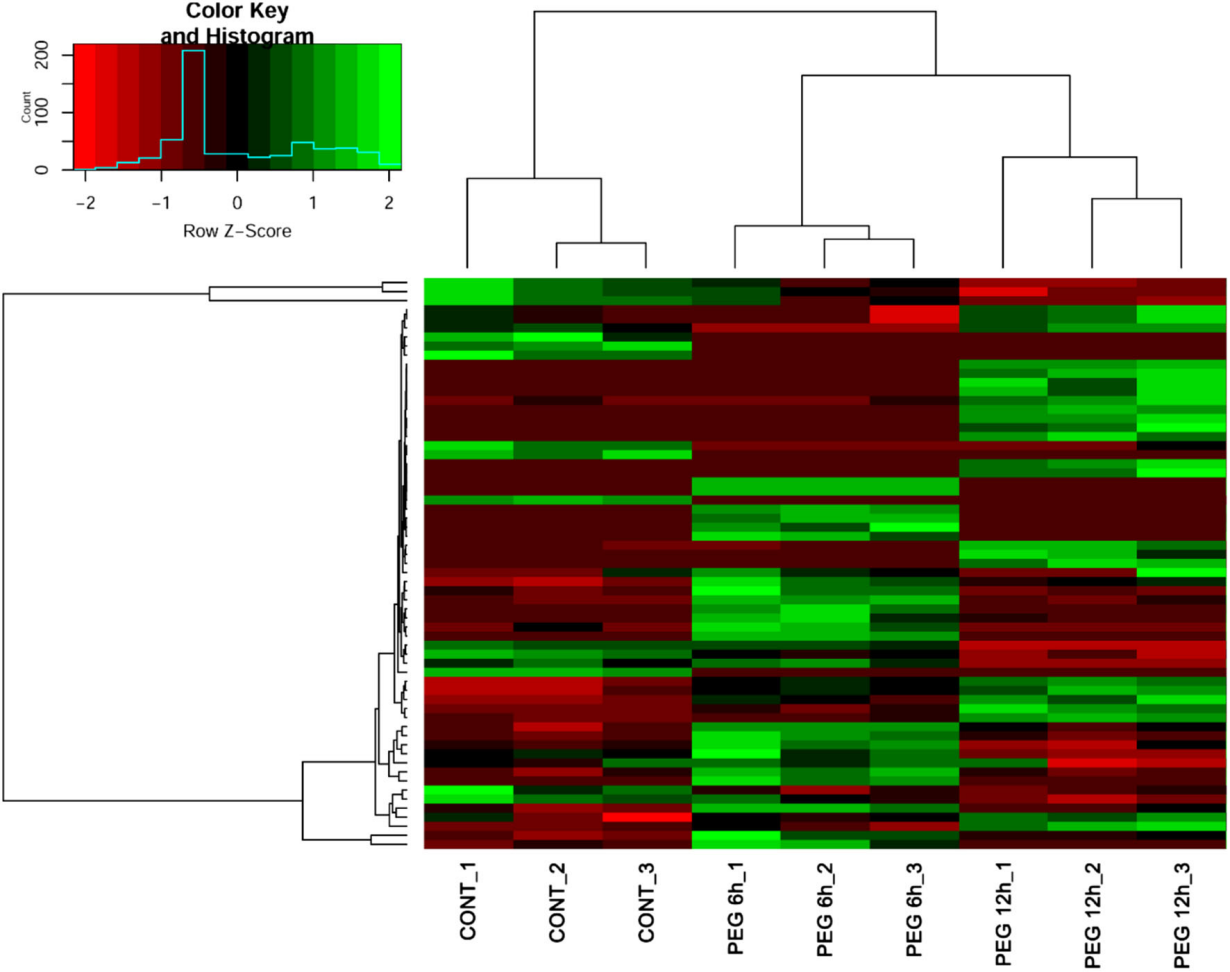
Heat map of the RNA-Seq-based transcriptome of *Populus davidiana* after 10% PEG treatment. The Heat map shows significant differences in the clustering of gene expression in response to 10% polyethylene glycol-induced drought stress in control, 6 h, and 12 h treatments

Figure 1 shows an overall profile of gene expression in response to drought stress induced by 10% PEG. In order to generate the transcriptome data, triplicates of leaf samples were collected for total RNA extraction from un-treated control and 10% PEG-treated plants grown on MS. These samples were used for cDNA library generation and then sequenced using Illumina high-throughput sequencing. An average of 58.5 million reads were generated from control plants and after 6 and 12 h of treatment with 10% PEG by using the genome of *P. trichocarpa* as a reference. The transcriptomic raw sequence data were released to the public through the National Agricultural Biotechnology Information Center (NABIC) database (http://www.nabic.rda.go.kr) with Accession Number (NN-2568-000001).

The transcriptome information for *P. davidiana* is shown in Table 1. A total of 32,087 and 32,330 genes showed differential expression after 6 and 12 h of treatment with 10% PEG, respectively. Of these, 12,403 (39%) and 12,414 (39%), respectively, were successfully mapped and annotated using the *P. trichocarpa* reference from PopGenie and Phytozome. Among these DEGs, a total 404 genes recorded at 6 h after PEG treatment were identified as transcription factors (238 and 166 genes were up- and down-regulated, respectively), whereas at 12 h after treatment compared to 6 h, a total of total of 359 DEGs were identified as transcription factors (187 and 172 genes were up- and down-regulated, respectively—Supplementary Figure S1). Significant differences were observed between the two time points with respect to the number, level of expression, and type of responsive genes. In general, the change in transcript accumulation (either up- or down-regulated) was greater after 6 h of stress treatment, indicating that there is a rapid response to water shortage at the molecular level via transcriptional regulation.

**Table 1:** Drought stress-responsive genes of *P. davidiana* compared with *P. trichocarpa* annotation, and the numbers of transcription factors classified using GO terms

Group 1	Group 2	Genes				
		Total differentially expressed genes	Annotation matched	GO term matched	Transcription factor	
					Up-regulated	Down-regulated
Control	6 h after PEG treatment	32,087	12,403 (39%)	5252 (42%)	404 (8%)	
					238	166
6 h after PEG treatment	12 h after PEG treatment	32,330	12,414 (38%)	5314 (43%)	359 (7%)	
					187	172

### Expression pattern of transcription factors

**Fig. 2:**
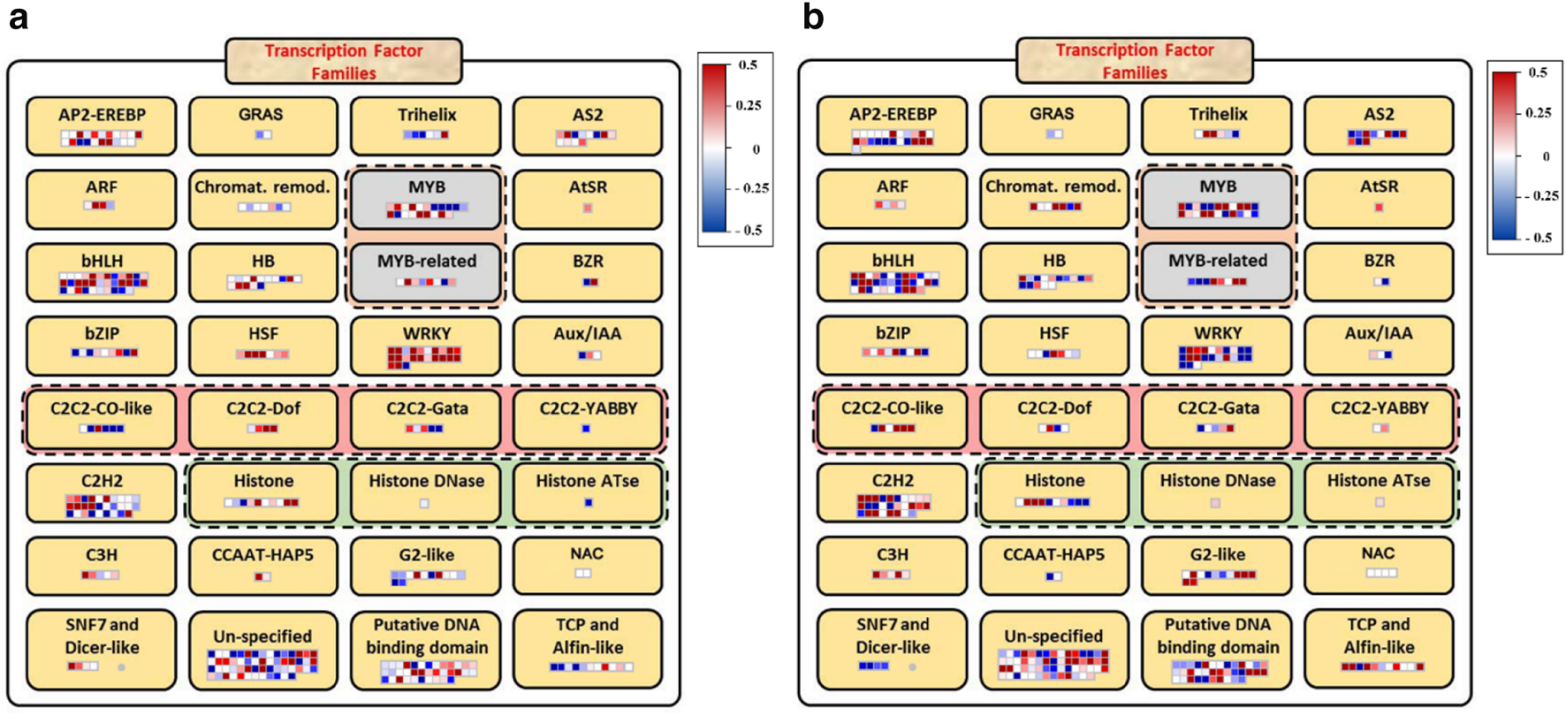
MapMan analysis of DEGs encoding transcription factors induced in response to drought stress induced by polyethylene glycol (PEG). All transcription factors among the differentially expressed genes were analyzed for functional classification using MapMan. The image shows an overview of the major transcription factors families regulated by 10% PEG after **a** 6 h and **b** 12 h of PEG treatment

Our results showed similar transcript expression profiles in various transcription factors at different time points following stress treatment. Detailed analysis of the data revealed that the DEGs contained various TFs families, including WRKY, MYB, bHLH, AP2-AREBP, C2C2-CO-like, C2C2-Dof, and C2H2. In addition, certain unspecified and putative DNA binding domain TFs were identified. Interestingly, in a comparison of two time points (6 and 12 h) after drought induction, most of the genes in the WRKY, MYB, and AP2-EREBP TF families, which play major roles in the response to drought and other abiotic stresses in other *Populus* species (Campbell 2010; Jiang et al. 2014), were up-regulated at 6 h but down-regulated at 12 h. However, some genes in the same TF groups (WRKY, MYB, and AP2-EREBP) remained up-regulated even at 12 h after drought induction. Similar transcript accumulation has observed in AS2, SNF7, and Dicer-like TFs. In contrast, the C2C2-CO-like, G2-like, and TCP TFs were down-regulated at 6 h but up-regulated at 12 h (Fig. 2).

### Expression pattern of transcription factors in *P. davidiana*

**Fig. 3:**
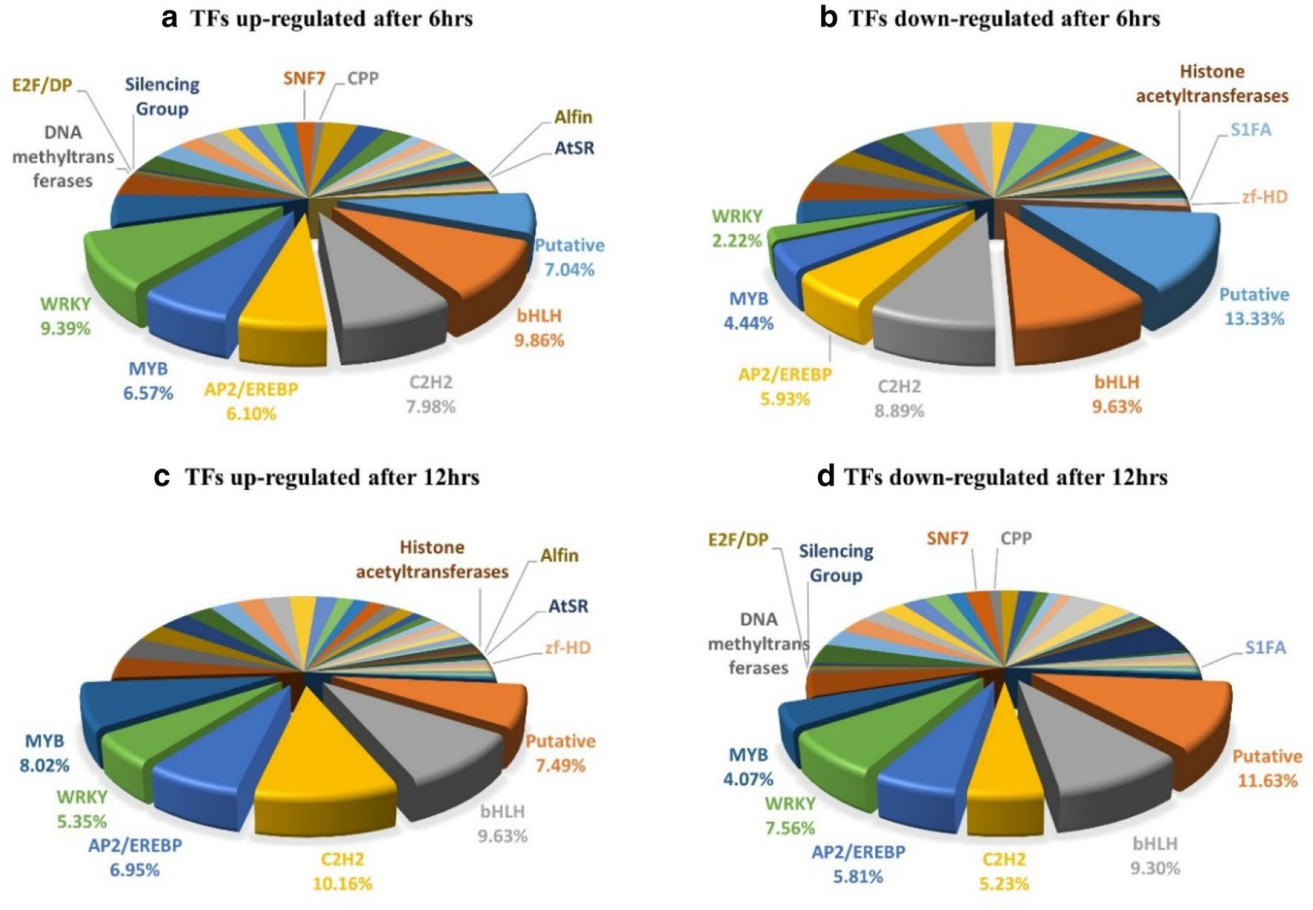
Expression patterns of various transcription factor (TF) groups in response to osmotic stress. The basic helix-loop-helix (bHLH) TFs were the major group of up-regulated transcripts after 6 h of polyethylene glycol (PEG) treatment, followed by the WRKY and C2H2 (**a**). The putative DNA-binding domain and C2H2 TFs comprised the major group of down-regulated transcripts at 6 h after PEG treatment (**b**); however, their expression had increased markedly after 12 h, along with that of C2H2, MYB, and AP2/EREBP (**c**). At this latter time point, the putative DNA-binding domain, bHLH, and WRKY TFs were among the down-regulated TFs (**d**)

To investigate the detailed expression pattern of TF families, a pie chart was generated using a total of 404 TFs expressed at 6 h (238 and 166 genes up- and down-regulated, respectively) and 359 TFs expressed at 12 h (187 and 172 genes up- and down-regulated) after 10% PEG treatment (Fig. 3). The results were similar to those obtained using MapMan analysis, with expression of bHLH, MYB, WRKY, C2H2 and AP2/EREBP domain-containing TFs being commonly up- and down-regulated at both time points. These major TFs accounted for almost half (45%) of all TFs from the total DEGs in response to PEG-induced drought stress.

Interestingly, bHLH TFs consistently showed up- and down-regulation of transcript levels, regardless of the time point analyzed. This result indicates that bHLH TFs may be involved in multiple signal pathways in adaptation to drought, as reviewed in (Castilhos et al. 2014). A list of the most up- and down-regulated TFs at 6 and 12 h after drought induction are presented in Supplementary Tables S1 and S2, respectively. In detail, the expression of a drought-responsive transcriptomic element (POPTR_0001s35280) homologous to AS2 domain-containing TFs was highest, being increased by more than 16-fold, followed by that of the PHOR1, WRKY, bHLH, AP2/EREBP, and MYB analogs. In contrast, TFs related to C2H2 TFs (POPTR_0010s13400) were down-regulated by more than 8 times, followed by transcripts related to C2C2-CO-like, ARR, bZIP, and putative DNA binding domain groups of TFs.

After 12 h of stress treatment, expression of bHLH (POPTR_0015s14070) TFs was increased by more than 32-fold, followed by C2H2, C2C2(Zn)-GATA, and GARP-G2-like TFs. At the same time point, TFs related to the Trihelix (POPTR_0012s11820) group were found to be down-regulated by more than 9.5 times, followed by members of the MADS box, C2H2, AP2-EREBP, and SET-domain-containing groups of TFs. Among the small groups of TFs, Alfin (POPTR_0016s12420) and AtSR (POPTR_0010s15160) were up-regulated after both 6 and 12 h in response to drought stress treatment. In contrast, S1FA TFs were down-regulated at both time points. In addition, transcript accumulation of SNF7, CPP, DNA methyltransferases, E2F/DP, and silencing TFs was increased at the early time point (6 h), but decreased as time progressed (12 h). Although histone acetyltransferases (POPTR_0015s10220) and zf-HD (POPTR_0019s03790) were down-regulated at 6 h, they were up-regulated at 12 h. Some of these TFs, such as SNF7, CPP, and E2F/DP, have been reported as sub-categories in response to heat stress in *Arabidopsis* and rice (Ueda et al. 2012). Taken together, these results indicate the diverse roles of these TFs in transcriptional regulation of key genes in drought stress response.

### Characterization of transcription factors in *P. davidiana*

In a comparison of previous studies on other *Populus* species, TFs such as C2H2, NAC, bHLH, WRKY, and AP2/EREBP were found to be commonly expressed in response to drought stress. In order to determine specific *P. davidiana* TF characteristics, raw sequences of RNA-seq reads were analyzed by IGV software using the *P. trichocarpa* genome as a reference. For a detailed analysis, three genes (*PtrZFP64*, *PtrZFP99*, and *PtrZFP103*) from C2H2 TFs, which were reported by Liu et al. (2015) to be involved in the drought response in *P. trichocarpa* and highly up- or down-regulated in the present study, were selected. One of these, *PtrZFP103* (POPTR_0018s10230), showed an obvious difference in expression pattern between *P. trichocarpa* and *P. davidiana*. According to the study, expression of *PtrZFP103* was not altered in *P. trichocarpa* leaves after drought stress. However, the expression level of this gene was markedly reduced in *P. davidiana*. This result indicates the possibility of sequence differences in certain genes among *Populus* species and that these differences lead to differential expression of genes in response to stress. To verify differences in gene sequences, transcript isoforms of *P. davidiana* were compared with those of the *P. trichocarpa* genome. We identified 11 sequence differences and confirmed the exact sequence of *P. davidiana* (Supplementary Table 3). This sequence was used in further analyses.

### Quantitative real-time PCR validation and SNP confirmation

**Fig. 4:**
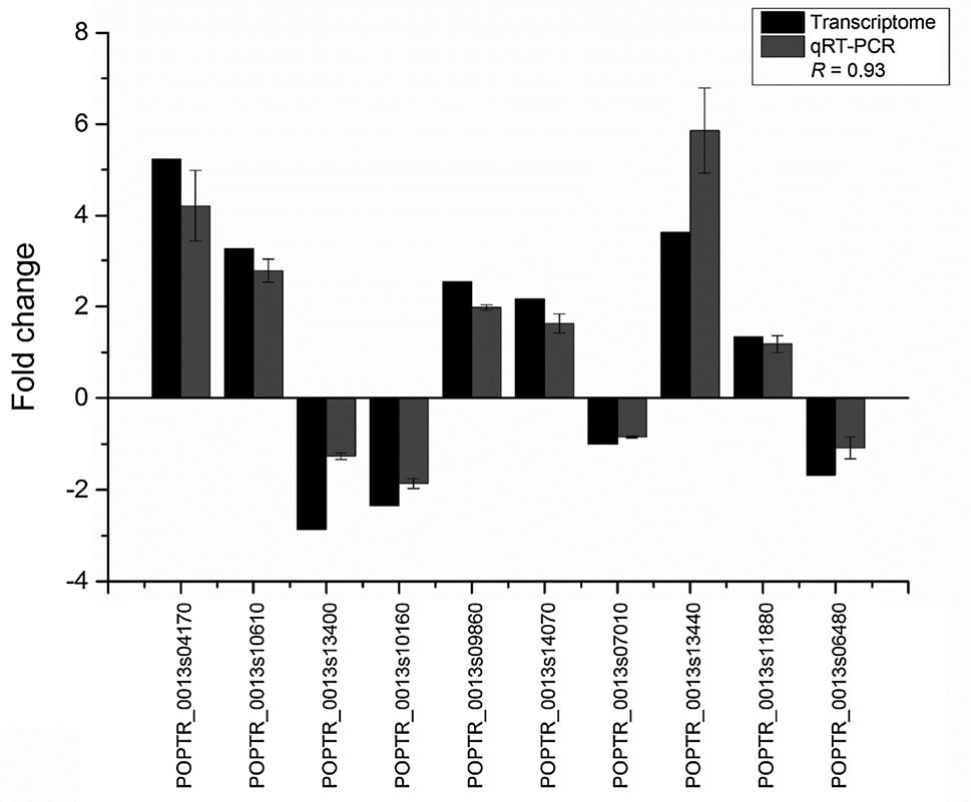
Quantitative real-time PCR validation of RNA-Seq results for selected genes. Ten differentially expressed genes that showed significant fold changes in their expression levels in transcriptome profiling (*black bars*) were selected, and their expression levels were validated by qRT-PCR (*dark gray*). The correlation coefficient *R* = 0.93 indicates a significant correlation between the results of qPCR and RNA-Seq

**Fig. 5:**
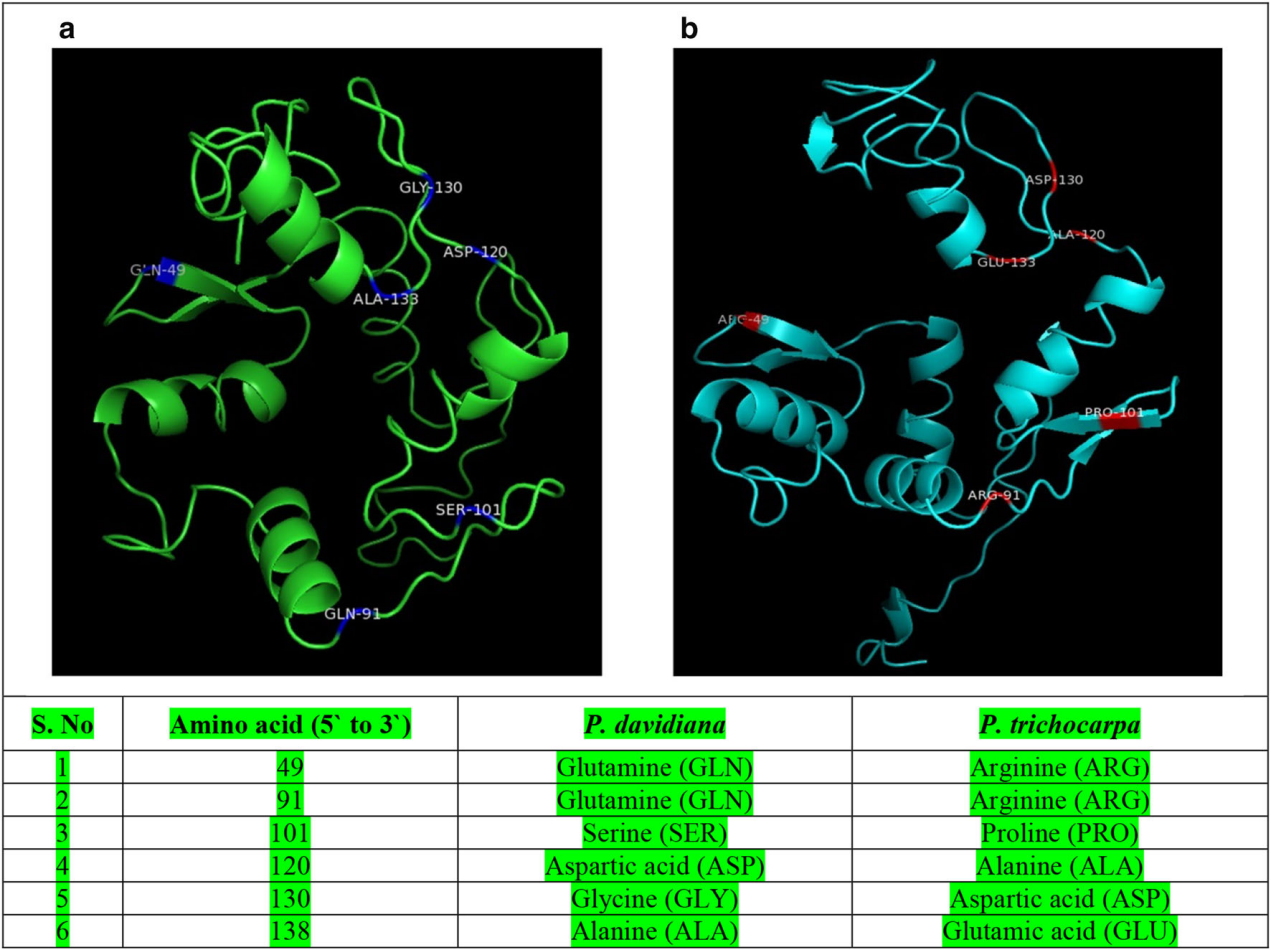
Comparison of predicted 3D protein structure of POPTR_0018s10230 from *P. davidiana* (**a**) and *P. trichocarpa* (**b**). The amino acid sequence of POPTR_0018s10230 was analyzed using the I-TASSER server, and the protein structure was generated using PyMOL software. Significant structural changes were attributable to changes in six amino acids

To validate PEG-mediated transcriptional changes in the transcriptome, a total of 10 representative genes, encoding transcription factors, i.e., putative DNA binding (POPTR_0013s04170), WRKY (POPTR_0016s10610), homeobox (POPTR_0014s09860), C2H2 (POPTR_0010s13400), *Arabidopsis* response regulator (POPTR_0014s10160), bHLH (POPTR_0015s14070), and MYB (POPTR_0017s11880) TFs, that showed significant changes in expression levels were selected for qRT-PCR analysis. The sequences of the primers used for qRT-PCR are listed in Supplementary Table 4. We observed significantly similar expression patterns of the transcripts in qRT-PCR and RNA-seq data as indicated by the correlation coefficient value (*R* = 0.93) shown in Fig. 4. Additionally, to identify representative *P. davidiana*-specific sequences (SNP), a gene that has been reported to be a drought response gene in *P. trichocarpa* (Liu et al. 2015) and showed significant changes in our transcriptome data was analyzed using I-Tasser server (Yang et al. 2015). Expression of this gene was substantially decreased after 6 h of 10% PEG treatment, and few SNP sequences were detected when compared to *P. trichocarpa*. Further, to verify the exact sequence of *P. davidiana,* the gene was cloned and sequenced. On the basis of this sequence, a predicted 3D protein structure was generated (Fig. 5). The predicted protein structures of this gene from the two *Populus* species showed significant differences. Notably, the amino acid sequence was different at six different positions (Fig. 5), resulting in a significantly different conformation of *P. davidiana*-specific protein. The resulting higher change in the expression of this gene following PEG treatment and the significant difference in the sequence/conformation of this protein compared to *P. trichocarpa* further validate its involvement in drought stress regulation.

## Discussion

Drought stress is a major problem worldwide because of global climate change. Numerous studies have investigated the drought stress response in crop plant species, and also in a few poplar species. Diverse physiological and biological regulations are involved in the response to drought stress. Among these, TFs, which regulate the activation or deactivation of other genes involved in upstream signal transduction, are good candidates for understanding the underlying mechanisms of the drought response. The role of TFs in abiotic stress responses, including drought stress, has been established in various plant species such as rice and *Arabidopsis* by using molecular techniques, including microarray analysis and RNA-seq-mediated transcriptome analysis (Fowler and Thomashow 2002; Nakashima et al. 2009; Rabbani et al. 2003; Yamaguchi-Shinozaki and Shinozaki 2006). However, scientific information, particularly that for TFs in the response of perennial forest trees to various abiotic stresses, including drought, is still largely lacking. In the present study, we carried out a transcriptome analysis of *P. davidiana* after application of 10% PEG to investigate the diverse changes in TFs mediated by drought stress.

In order to determine the total number and types of DEGs, the transcriptomic data were subjected to quality control screening, and DEGs with a cutoff *p* value <0.05 were selected for further studies (Table 1). Of the total DEGs, approximately 40% were successfully mapped and annotated using the *P. trichocarpa* reference genome data from PopGenie and Phytozome. A relatively low percentage of TFs was detected in our study compared to that detected for *Arabidopsis* and rice. This can be attributed to the lack of information on poplar species, for which the functions of many genes have not been predicted. In addition, the numbers of TFs among DEGs were relatively higher at 6 h compared to 12 h after PEG treatment. This indicates that plants respond rapidly to drought stress at the molecular level and initiate transcriptional regulation. Once plants detect abiotic stress, such as cold, salt, and drought, diverse of pathways are induced as a means of defense and TFs are among the key factors comprising a regulating defense system. There is a significant overlap between gene expression patterns induced by different types of stresses (Chen et al. 2002; Durrant et al. 2000). TFs families, including WRKY, MYB, bHLH, AP2-AREBP, C2C2-CO-like, C2C2-Dof, and C2H2, well-known TFs that are highly related to the drought responses in *Arabidopsis* (Saibo et al. 2009), rice (Moumeni et al. 2011) and soybean (Pereira et al. 2011), were most abundantly observed, and these TFs were primarily involved in drought stress response at 6 and 12 h. Among these, WRKY, MYB, and AP2-EREBP TFs, which are known to be drought-responsive TFs in other *Populus* species were up-regulated at 6 h but down-regulated at 12 h. However, some genes (e.g., POPTR_0013s04170) from the same TF groups remained up-regulated even at 12 h. In contrast, the C2C2-CO-like, G2-like, and TCP TFs were down-regulated at 6 h but up-regulated at 12 h (Fig. 2). These observations indicate that there is a significant crosstalk between TFs at the cellular level. For example, after 6 h of stress treatment, some members of the WRKY and HSF groups of TFs were up-regulated, whereas others were down-regulated. The WRKY signaling cascade is involved in both biotic and abiotic stress responses. Many studies have now established that these genes transcriptionally co-regulate responses to various types of stress. The magnitude of the role of WRKY TFs is demonstrated by the rice *OsWRKY13* gene, which regulates the expression of more than 500 stress-responsive genes (Qiu et al. 2008). Overexpression of the *PtoWRKY60* gene in *Populus tomentosa* resulted in a marked increase in the expression of the defense genes *PR5.1, PR5.2, PR5.5,* and *CPR5*. Additionally, overexpression of the salicylic acid-inducible *PtrWRKY73* from *P. trichocarpa* enhanced the resistance of *Arabidopsis* plants to *Pst*DC3000.

In addition, AP2/EREBP and bHLH TFs groups showed strong differential expression at both the time points examined in the present study. According to the Plant Transcription Factor Database (plntfdb.bio.uni-potsdam.de/v3.0/), 139 loci have been identified for the AP2/EREBP family of TFs in *Arabidopsis*, 163 and 138 in *Oryza sativa* (*japonica* and *indica,* respectively), 204 in *Zea mays*, 338 in *Glycine max*, and 209 in *P. trichocarpa*. Similarly, 225 loci have been identified for the bHLH family of TFs in *Arabidopsis*, 548 in *Glycine max*, 211 and 169 for *Oryza sativa* (*japonica* and *indica,* respectively), 388 in *Zea mays*, and 379 in *P. trichocarpa.* These data indicate that AP2/EREBP and bHLH TFs are conserved in the plant kingdom, and previous research has shown that these TFs are part of a gene regulatory network for integrating metabolic, hormonal, sugar, and redox signaling in response to abiotic stresses such as cold and drought (Dietz et al. 2010). For example, the *Arabidopsis* AP2/EREBP-type TF, *AtERF7*, plays an important role in ABA response by interacting with protein kinase PKS3, a global regulator of ABA responses. *AtERF7* binds to the GCC box and acts as a repressor of gene transcription. The stomatal guard cells of *Arabidopsis* plants overexpressing *AtEFR7* show less sensitivity to ABA, and these plants have increased transpiration (Song et al. 2005). Furthermore, one of the bHLH family TFs in *Populus*, PebHLH35 from *Populus euphratica,* has been reported as important gene in the drought tolerance response by regulating stomatal development and photosynthesis in *Arabidopsis* (Dong et al. 2014b).

*P. davidiana* transcriptomic elements homologous to MYB TFs were also a major part of the genes examined in the present study. Functional characterization of *P. tomentosa* R2R3-MYB transcription factor *PtoMYB216* revealed its involvement in lignin biosynthesis. A 1.8-kb promoter sequence of this gene fused to the GUS coding sequence and introduced into *Arabidopsis* showed that GUS expression was restricted to tissues undergoing secondary cell wall formation. Overexpression of this gene enhanced the expression of lignin biosynthetic pathway genes and resulted in lignin deposition, even in cells that are normally unlignified (Tian et al. 2013). In rice, overexpression of the *OsMYB48*-*1* transcription factor enhanced tolerance to drought and salt stress. *OsMYB48*-*1* was strongly induced by PEG, ABA, H_2_O_2_, and dehydration, whereas it was slightly induced by salinity and cold stress. Overexpression of *OsMYB48*-*1* in rice significantly increases tolerance to drought and salt stress induced by mannitol, PEG, and NaCl, and also regulates the expression of ABA biosynthesis genes (*OsNCED4* and *OsNCED5*) (Xiong et al. 2014). Collectively, the transcriptomic data generated in this study serve as a good platform for further studies investigating and incorporating biotic and abiotic stress tolerance in plants.

TFs related to MYB, AP2-EREBP, C2H2, C2C2-CO-like, trihelix, and NAC families are good candidates for plant functional genomic studies. Among these TFs, trihelix and C2H2 TFs have been reported to be functionally involved in drought response in poplar (Liu et al. 2015; Weng et al. 2012). Interestingly, three genes (*POPTR_0016s10460*, *POPTR_0016s10480*, and *POPTR_0018s10230*) of the C2H2 TF family, reported to be drought response genes in *P. trichocarpa,* were detected in our transcriptome data set containing the 10 most down-regulated and 10 most up-regulated genes. As reported by (Zhang et al. 2015), two species of *Populus* (*P. tremula* and *P. tremuloides*) growing in different ecological circumstance showed genetic diversity. In this regard, to identify any genetic difference in *P. davidiana,* the raw RNA-seq reads of three genes were analyzed with IGV software using the *P. trichocarpa* genome as a reference. Among these, POPTR_0018s10230 showed a substantial difference in sequence between *P. davidiana* and *P. trichocarpa*, and the exact sequence of *P. davidiana* was confirmed by clone-based sequencing. The sequence of genes affects the structure of proteins, and such structural changes result in functional changes in proteins (Kishor et al. 2015).

Accordingly, 3D protein structures were generated as shown in Fig. 5. Comparison of the sequences of *P. davidiana* and *P. trichocarpa* revealed a difference in six amino acids. Of these, the proline at position 101 was changed to serine and was associated with a significant structural alteration. Proline is known to function in maintaining structural stability and in abiotic stress response, particularly that to drought and salt stress. For example, proline accumulation was increased fourfold under osmotic stress in a resistant maize cultivar, and proline helps to stabilize subcellular structures in the control of osmolytes. Additionally, exogenous application of proline affects plant growth and photosynthesis, and improves the drought tolerance response (Hayat et al. 2012; Jibran et al. 2015; Rai 2002).

The results of the present study suggest that there is genetic diversity between *P. davidiana* and *P. trichocarpa* and that changes in a few sequences lead to different roles of protein in response to drought stress. On the basis of this information, further biological studies using knock out/down and overexpression mutant plants of these two poplar species would provide further information on the role of this gene in response to drought stress. Taken together, the results of this study of *P. davidiana* TFs under drought stress will expand our knowledge of drought stress-responsive TFs, particularly with respect to how they regulate an intricate system at the cellular level, involving proteins responsible for upstream signal transduction and transcriptional regulation, and also suggest a rich resource for discovering critical genes related to the drought stress response in *P. davidiana.*

## Electronic supplementary material

Below is the link to the electronic supplementary material.

Supplementary material 1 (DOCX 163 kb)

Supplementary material 2 (DOCX 17 kb)

Supplementary material 3 (DOCX 15 kb)

Supplementary material 4 (DOCX 14 kb)

Supplementary material 5 (DOCX 14 kb)

## References

[CR1] Avramova V, Abdelgawad H, Asard H, Beemster GT (2014). The growth zone of maize leaves subjected to drought stress offers unique possibilities to confirm transcriptome analysis with cellular, physiological and biochemical measurements. Commun Agric Appl Biol Sci.

[CR2] Bedada G (2014). Transcriptome sequencing of two wild barley (*Hordeum spontaneum* L.) ecotypes differentially adapted to drought stress reveals ecotype-specific transcripts. BMC Genom.

[CR3] Campbell AS (2010) Drought response of *Populus* transformed with stress response transcription factors. Master's Thesis, University of Tennessee. http://trace.tennessee.edu/utk_gradthes/690

[CR4] Castilhos G, Lazzarotto F, Spagnolo-Fonini L, Bodanese-Zanettini MH, Margis-Pinheiro M (2014). Possible roles of basic helix-loop-helix transcription factors in adaptation to drought. Plant Sci.

[CR5] Chen WQ (2002). Expression profile matrix of *Arabidopsis* transcription factor genes suggests their putative functions in response to environmental stresses. Plant Cell.

[CR6] Chen L, Zhang YY, Ren YY, Xu JC, Zhang ZY, Wang YW (2012). Genome-wide identification of cold-responsive and new microRNAs in *Populus tomentosa* by high-throughput sequencing. Biochem Biophy Res Commun.

[CR7] Chen J, Tian Q, Pang T, Jiang L, Wu R, Xia X, Yin W (2014). Deep-sequencing transcriptome analysis of low temperature perception in a desert tree *Populus euphratica*. BMC Genom.

[CR8] Coder KD (1999) Drought damage to trees. University of Georgia Daniel B. Warnell School of Forest Resources Extension publication. http://www.forestry.uga.edu/efr

[CR9] Dietz KJ, Vogel MO, Viehhauser A (2010). *AP2/EREBP* transcription factors are part of gene regulatory networks and integrate metabolic, hormonal and environmental signals in stress acclimation and retrograde signalling. Protoplasma.

[CR10] Dong Y, Wang C, Han X, Tang S, Liu S, Xia X, Yin W (2014). A novel bHLH transcription factor *PebHLH35* from *Populus euphratica* confers drought tolerance through regulating stomatal development, photosynthesis and growth in *Arabidopsis*. Biochem Biophys Res Commun.

[CR11] Dong Y, Wang CP, Han X, Tang S, Liu S, Xia XL, Yin WL (2014). A novel bHLH transcription factor PebHLH35 from *Populus euphratica* confers drought tolerance through regulating stomatal development, photosynthesis and growth in *Arabidopsis*. Biochem Bioph Res Commun.

[CR12] Durrant WE, Rowland O, Piedras P, Hammond-Kosack KE, Jones JDG (2000). cDNA-AFLP reveals a striking overlap in race-specific resistance and wound response gene expression profiles. Plant Cell.

[CR13] Flicek P (2014). Ensembl 2014. Nucleic Acids Res.

[CR14] Fowler S, Thomashow MF (2002). *Arabidopsis* transcriptome profiling indicates that multiple regulatory pathways are activated during cold acclimation in addition to the CBF cold response pathway. Plant Cell.

[CR15] Gong L (2015). Transcriptome profiling of the potato (*Solanum tuberosum* L.) plant under drought stress and water-stimulus conditions. PLoS One.

[CR16] Gu RS, Fonseca S, Puskas LG, Hackler L, Zvara A, Dudits D, Pais MS (2004). Transcript identification and profiling during salt stress and recovery of *Populus euphratica*. Tree Physiol.

[CR17] Hamanishi ET, Campbell MM (2011). Genome-wide responses to drought in forest trees. Forestry.

[CR18] Hayat S, Hayat Q, Alyemeni MN, Wani AS, Pichtel J, Ahmad A (2012). Role of proline under changing environments: a review. Role of proline under changing environments: a review Plant Signal Behav.

[CR19] Hu RB, Qi GA, Kong YZ, Kong DJ, Gao QA, Zhou GK (2010). Comprehensive analysis of NAC domain transcription factor gene family in *Populus trichocarpa*. BMC Plant Biol.

[CR20] Huang L, Zhang F, Zhang F, Wang W, Zhou Y, Fu B, Li Z (2014). Comparative transcriptome sequencing of tolerant rice introgression line and its parents in response to drought stress. BMC Genom.

[CR21] Hukin D, Cochard H, Dreyer E, Le Thiec D, Bogeat-Triboulot MB (2005). Cavitation vulnerability in roots and shoots: does *Populus euphratica* Oliv., a poplar from arid areas of Central Asia, differ from other poplar species?. J Exp Bot.

[CR22] Hussain A (2016). Nitric oxide mediated transcriptome profiling reveals activation of multiple regulatory pathways in *Arabidopsis thaliana*. Front Plant Sci.

[CR23] Jiang YZ (2014). Genome-wide identification and characterization of the *Populus* WRKY transcription factor family and analysis of their expression in response to biotic and abiotic stresses. J Exp Bot.

[CR24] Jibran R (2015). Staying green postharvest: how three mutations in the *Arabidopsis* chlorophyll b reductase gene NYC1 delay degreening by distinct mechanisms. J Exp Bot.

[CR25] Jordan WR, Ritchie JT (1971). Influence of soil water stress on evaporation, root absorption, and internal water status of cotton. Plant Physiol.

[CR26] Kido EA (2012). New insights in the sugarcane transcriptome responding to drought stress as revealed by superSAGE. TheScientificWorldJournal.

[CR27] Kishor PBK, Kumari PH, Sunita MSL, Sreenivasulu N (2015) Role of proline in cell wall synthesis and plant development and its implications in plant ontogeny. Front Plant Sci 6 doi: 10.3389/fpls.2015.00544 (**ARTN 544**) 10.3389/fpls.2015.00544 PMC4507145 26257754

[CR28] Kozlowski TT, Pallardy SG (2002) Acclimation and adaptive responses of woody plants to environmental stresses. Bot Rev 68:270–334. doi:10.1663/0006-8101(2002)068[0270:Aaarow]2.0.Co;2

[CR29] Kumar S, Kalra S, Singh B, Kumar A, Kaur J, Singh K (2016). RNA-Seq mediated root transcriptome analysis of Chlorophytum borivilianum for identification of genes involved in saponin biosynthesis. Funct Integr Genom.

[CR30] Kwon S-H, Kwon H-K, Kim W, Noh EW, Kwon M, Choi YI (2014). Identification of salt and drought inducible glutathione S-transferase genes of hybrid poplar. J Plant Biotechnol.

[CR31] Le DT (2012). Differential gene expression in soybean leaf tissues at late developmental stages under drought stress revealed by genome-wide transcriptome analysis. PloS One.

[CR32] Lendzemo V (2009). The arbuscular mycorrhizal host status of plants can not be linked with the Striga seed-germination-activity of plant root exudates. J Plant Dis Prot.

[CR33] Li B, Qin Y, Duan H, Yin W, Xia X (2011). Genome-wide characterization of new and drought stress responsive microRNAs in *Populus euphratica*. J Exp Bot.

[CR34] Li BS, Qin YR, Duan H, Yin WL, Xia XL (2011). Genome-wide characterization of new and drought stress responsive microRNAs in *Populus euphratica*. J Exp Bot.

[CR35] Liu QG, Wang ZC, Xu XM, Zhang HZ, Li CH (2015). Genome-wide analysis of C2H2 Zinc-finger family transcription factors and their responses to abiotic stresses in poplar (*Populus trichocarpa*). PLoS One.

[CR36] Ma XD, Ma JC, Fan D, Li CF, Jiang YZ, Luo KM (2016) Genome-wide identification of TCP family transcription factors from *Populus euphratica* and their involvement in leaf shape regulation. Sci Rep Uk 6. doi: 10.1038/srep32795 (**ARTN 32795**) 10.1038/srep32795 PMC5015053 27605130

[CR37] Moumeni A (2011). Comparative analysis of root transcriptome profiles of two pairs of drought-tolerant and susceptible rice near-isogenic lines under different drought stress. BMC Plant Biol.

[CR38] Muscolo A, Sidari M, Anastasi U, Santonoceto C, Maggio A (2014). Effect of PEG-induced drought stress on seed germination of four lentil genotypes J Plant Interact.

[CR39] Nakashima K, Ito Y, Yamaguchi-Shinozaki K (2009). Transcriptional regulatory networks in response to abiotic stresses in *Arabidopsis* and grasses. Plant Physiol.

[CR40] Okay S, Derelli E, Unver T (2014). Transcriptome-wide identification of bread wheat WRKY transcription factors in response to drought stress. Mol Genet Genom MGG.

[CR41] Osakabe Y, Osakabe K, Shinozaki K, Tran LSP (2014). Response of plants to water stress. Front Plant Sci.

[CR42] Paul A, Jha A, Bhardwaj S, Singh S, Shankar R, Kumar S (2014). RNA-seq-mediated transcriptome analysis of actively growing and winter dormant shoots identifies non-deciduous habit of evergreen tree tea during winters. Sci Rep.

[CR43] Pereira SS (2011). Transcription factors expressed in soybean roots under drought stress. Genet Mol Res.

[CR44] Qiu DY, Xiao J, Xie WB, Liu HB, Li XH, Xiong LZ, Wang SP (2008). Rice gene network inferred from expression profiling of plants overexpressing *OsWRKY13*, a positive regulator of disease resistance. Mol Plant.

[CR45] Qiu Q (2011). Genome-scale transcriptome analysis of the desert poplar *Populus euphratica*. Tree Physiol.

[CR46] Rabbani MA (2003). Monitoring expression profiles of rice genes under cold, drought, and high-salinity stresses and abscisic acid application using cDNA microarray and RNA get-blot analyses. Plant Physiol.

[CR47] Rai VK (2002). Role of amino acids in plant responses to stresses. Biol Plant.

[CR48] Saibo NJM, Lourenco T, Oliveira MM (2009). Transcription factors and regulation of photosynthetic and related metabolism under environmental stresses. Ann Bot Lond.

[CR49] Si J, Zhou T, Bo W, Xu F, Wu R (2014). Genome-wide analysis of salt-responsive and novel microRNAs in *Populus euphratica* by deep sequencing. BMC Genet.

[CR50] Song CP, Agarwal M, Ohta M, Guo Y, Halfter U, Wang P, Zhu JK (2005). Role of an *Arabidopsis AP2/EREBP*-type transcriptional repressor in abscisic acid and drought stress responses. Plant Cell.

[CR51] Tang S (2013). *Populus euphratica*: the transcriptomic response to drought stress. Plant Mol Biol.

[CR52] Tang S (2013). *Populus euphratica*: the transcriptomic response to drought stress. Plant Mol Biol.

[CR53] Tian Q, Wang X, Li C, Lu W, Yang L, Jiang Y, Luo K (2013). Functional characterization of the poplar *R2R3*-*MYB* transcription factor *PtoMYB216* involved in the regulation of lignin biosynthesis during wood formation. PLoS One.

[CR54] Trapnell C, Pachter L, Salzberg SL (2009). TopHat: discovering splice junctions with RNA-Seq. Bioinformatics.

[CR55] Trapnell C (2010). Transcript assembly and quantification by RNA-Seq reveals unannotated transcripts and isoform switching during cell differentiation. Nat Biotechnol.

[CR56] Ueda K, Matsuura H, Yamaguchi M, Demura T, Kato K (2012). Genome-wide analyses of changes in translation state caused by elevated temperature in *Oryza sativa*. Plant Cell Physiol.

[CR57] Wang H, Zhao S, Gao Y, Yang J (2017). Characterization of Dof transcription factors and their responses to osmotic stress in poplar (*Populus trichocarpa*). PLoS One.

[CR58] Weng H, Yoo CY, Gosney MJ, Hasegawa PM, Mickelbart MV (2012). Poplar GTL1 Is a Ca2 +/calmodulin-binding transcription factor that functions in plant water use efficiency and drought tolerance. PLoS One.

[CR59] Xiong H (2014). Overexpression of *OsMYB48*-*1*, a novel MYB-related transcription factor, enhances drought and salinity tolerance in rice. PLoS One.

[CR60] Yamaguchi-Shinozaki K, Shinozaki K (2006). Transcriptional regulatory networks in cellular responses and tolerance to dehydration and cold stresses. Annu Rev Plant Biol.

[CR61] Yan DH, Fenning T, Tang S, Xia X, Yin W (2012). Genome-wide transcriptional response of *Populus euphratica* to long-term drought stress. Plant Sci.

[CR62] Yang JY, Yan RX, Roy A, Xu D, Poisson J, Zhang Y (2015). The I-TASSER Suite: protein structure and function prediction. Nat Methods.

[CR63] Zhang W (2014). Transcriptome sequencing of transgenic poplar (*Populus* × *euramericana* ‘Guariento’) expressing multiple resistance genes. BMC Genet.

[CR64] Zhang C et al. (2015) Genetic diversity in aspen and its relation to arthropod abundance. Front Plant Sci 5. doi: 10.3389/fpls.2014.00806 (**ARTN 806**) 10.3389/fpls.2014.00806 PMC4309117 25674097

