# Supplementary material for: Analysis of transcription factors among differentially expressed genes induced by drought stress in Populus davidiana
Source: 3 Biotech. 2017 Jun 30;7(3):209. doi: 10.1007/s13205-017-0858-7 (PMC5493580; doi:10.1007/s13205-017-0858-7)
Supplement: Supplementary file 1 — Supplementary material 1 (DOCX 163 kb) [file 13205_2017_858_MOESM1_ESM.docx]

A


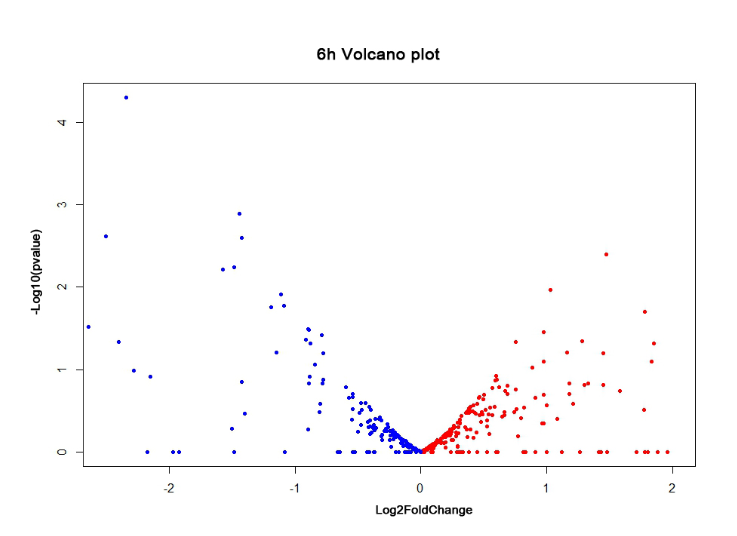


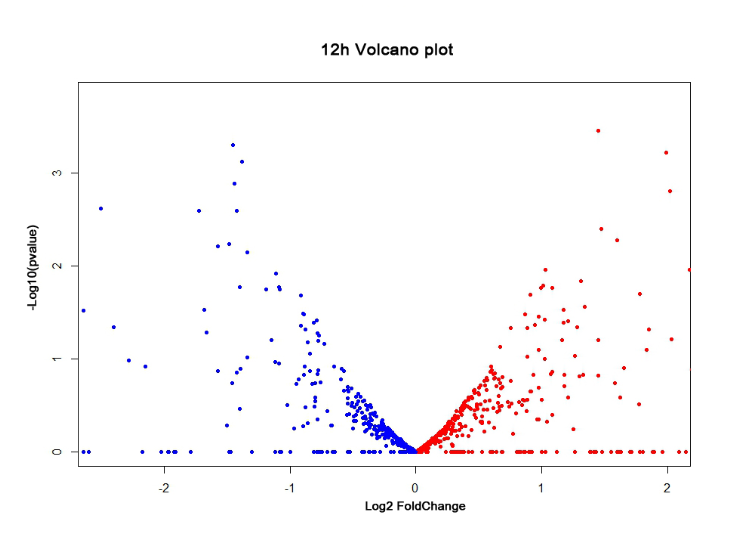


B

**Figure S1. Volcano graph of differentially expressed transcription factors.** The volcano plot shows DEGs of TFs) after 6 h of treatment (A) and 12 h of treatment (B).
