# Supplementary material for: Analysis of transcription factors among differentially expressed genes induced by drought stress in Populus davidiana
Source: 3 Biotech. 2017 Jun 30;7(3):209. doi: 10.1007/s13205-017-0858-7 (PMC5493580; doi:10.1007/s13205-017-0858-7)
Supplement: Supplementary file 2 — Supplementary material 2 (DOCX 17 kb) [file 13205_2017_858_MOESM2_ESM.docx]

**Supplementary Table S1 List of Top-10 up and down-regulated TFs from DEGs of *P. davidiana* transcriptome after 6hrs of 10%PEG treatment**

| **Up-regulated TFs** | | | |
| --- | --- | --- | --- |
| **NO.** | **Accession** | **Log2** | **Transcription factor family** |
| 1 | POPTR_0013s04170 | 5.24 | Putative DNA binding domain |
| 2 | POPTR_0001s35280 | 4.06 | AS2 |
| 3 | POPTR_0001s22370 | 3.95 | PHOR1 |
| 4 | POPTR_0001s02520 | 3.69 | Putative DNA binding domain |
| 5 | POPTR_0012s11820 | 3.32 | Trihelix |
| 6 | POPTR_0016s10610 | 3.27 | WRKY |
| 7 | POPTR_0012s01370 | 2.79 | bHLH |
| 8 | POPTR_0018s08320 | 2.76 | AP2/EREBP |
| 9 | POPTR_0001s41920 | 2.74 | MYB |
| 10 | POPTR_0001s44150 | 2.66 | bHLH |

| **Down-regulated TFs** | | | |
| --- | --- | --- | --- |
| **NO.** | **Accession** | **Log2** | **Transcription factor family** |
| 1 | POPTR_0010s13400 | -2.86 | C2H2 |
| 2 | POPTR_0010s25830 | -2.5 | C2C2(Zn)-CO-like |
| 3 | POPTR_0018s10230 | -2.4 | C2H2 |
| 4 | POPTR_0014s10160 | -2.34 | ARR |
| 5 | POPTR_0017s06670 | -2.28 | C2C2(Zn)-CO-like |
| 6 | POPTR_0016s13350 | -2.17 | BZR |
| 7 | POPTR_0010s14530 | -2.15 | bZIP |
| 8 | POPTR_0010s19180 | -1.97 | AS2 |
| 9 | POPTR_0013s11660 | -1.97 | Putative DNA binding domain |
| 10 | POPTR_0018s08090 | -1.92 | C2C2(Zn)-GATA |
