# Supplementary material for: Analysis of transcription factors among differentially expressed genes induced by drought stress in Populus davidiana
Source: 3 Biotech. 2017 Jun 30;7(3):209. doi: 10.1007/s13205-017-0858-7 (PMC5493580; doi:10.1007/s13205-017-0858-7)
Supplement: Supplementary file 3 — Supplementary material 3 (DOCX 15 kb) [file 13205_2017_858_MOESM3_ESM.docx]

**Supplementary Table S2 List of Top-10 up and down-regulated transcription factors from DEGs of *P. davidiana* transcriptome after 12hrs of 10%PEG treatment**

| **Up-regulated TFs** | | | |
| --- | --- | --- | --- |
| **NO.** | **Accession** | **Log2** | **Transcription factor family** |
| 1 | POPTR_0015s14070 | 4.06 | bHLH |
| 2 | POPTR_0010s13400 | 3.63 | C2H2 |
| 3 | POPTR_0016s10460 | 3.61 | C2H2 |
| 4 | POPTR_0018s08090 | 3.3 | C2C2(Zn)-GATA |
| 5 | POPTR_0010s14530 | 2.75 | bZIP |
| 6 | POPTR_0016s07010 | 2.51 | PHOR1 |
| 7 | POPTR_0001s02530 | 2.28 | C2H2 |
| 8 | POPTR_0010s25830 | 2.28 | C2C2(Zn)-CO-like |
| 9 | POPTR_0012s14080 | 2.19 | bHLH |
| 10 | POPTR_0013s04170 | 2.17 | Putative DNA binding domain |

| **Down-regulated TFs** | | | |
| --- | --- | --- | --- |
| **NO.** | **Accession** | **Log2** | **Transcription factor family** |
| 1 | POPTR_0013s13340 | -3.42 | bHLH |
| 2 | POPTR_0012s11820 | -3.27 | Trihelix |
| 3 | POPTR_0010s24540 | -2.64 | MADS box |
| 4 | POPTR_0010s20740 | -2.6 | C2H2 |
| 5 | POPTR_0016s10480 | -2.02 | C2H2 |
| 6 | POPTR_0016s05360 | -1.96 | AP2/EREBP |
| 7 | POPTR_0018s02550 | -1.91 | WRKY |
| 8 | POPTR_0001s39150 | -1.79 | SET-domain |
| 9 | POPTR_0017s11880 | -1.72 | MYB |
| 10 | POPTR_0014s09860 | -1.68 | HB |
