# Supplementary material for: Analysis of transcription factors among differentially expressed genes induced by drought stress in Populus davidiana
Source: 3 Biotech. 2017 Jun 30;7(3):209. doi: 10.1007/s13205-017-0858-7 (PMC5493580; doi:10.1007/s13205-017-0858-7)
Supplement: Supplementary file 4 — Supplementary material 4 (DOCX 14 kb) [file 13205_2017_858_MOESM4_ESM.docx]

**Supplementary Table S3 Alignment of *P. davidiana*-specific POPTR_0015s06480 with *P. trichocarpa*.**

*P.trochocarpa*      ATGGATTTCCAACCAAACACCTCTCTACATCTAAGCCTACCAAGCAATCAACTAAACCTA 
*P.davidiana*        ATGGATTTTCAACCAAACACCTCTCTACATCTAAGCCTACCAAGCAATCAACTAAACCTA 
                   ******** *************************************************** 

*P.trochocarpa*      GAACTTGTACTCGAGCCATCCTCTTCTTCTTCATCATCACCTCATAGTCCGGCAGAACCT 
*P.davidiana*        GAACTTGTACTCGAGCCATCCTCTTCTTCTTCATCATCACCTCATAGTCCGGCAGAACCT 
                   ************************************************************ 

*P.trochocarpa*     CGAATTTTCTCATGCAACTACTGCCGAAGAAAGTTTTATAGCTCACAAGCTCTTGGGGGT 
*P.davidiana*       CGAATTTTCTCATGCAACTACTGCCAAAGAAAGTTTTATAGCTCACAAGCTCTTGGGGGT 
                   ************************* ********************************** 

*P.trochocarpa*      CACCAAAATGCTCATAAGCTTGAGAGAACCTTGGCCAAAAAGAGCCGAGAGATGAGTTCA 
*P.davidiana*        CACCAAAATGCTCATAAGCTTGAGAGAACCTTGGCCAAAAAGAGCCGAGAGATGAGTTCA 
                   ************************************************************ 

*P.trochocarpa*      TCCGTACGGGCTCATGGAAGATCGAACCCACGGTCAGGATCGTCTTCTTGCATGAGTGGG 
*P.davidiana*        TCCGTACGGGCTCATGGAAGATCGAACCCACAGTCTGGATCGTCTTCTTGCATGAGTGGG 
                   ******************************* *** ************************ 

*P.trochocarpa*     CCAAGCTTTCCTCGACATCATGAACCAGCCCTAGCAAGGTTCGAGCACCATGGACATGCT 
*P.davidiana*        TCAAGCTTTCCTAGACATCATGAGCCGGCCCTAGCAAGGTTCGAGCACCATGGACATGAT 
                    *********** ********** ** ******************************* * 

*P.trochocarpa*      GGTAGGTTTGTTGGTGACGCGAGCTATGACAGGACAGAGATGAATTATGGTTCCATAGAA 
*P.davidiana*        GGTAGGTTTGTTGGTGACGCGAGCTATGGCAGGACAGCGATGAATTATGGTTCCATAGAA 
                   **************************** ******** ********************** 

*P.trochocarpa*      GGTGTAGGGGGTTCTTGGTCCCTGGTATATAGAACAGAAAATGTTCAAGAAGAGTTGAGC 
*P.davidiana*        GGTGTAGGGGGTTCTTGGTCCCTGGTGTATAGAACCGAAAATGTTCAAGAAGAGTTGAGC 
                   ************************** ******** ************************ 

*P.trochocarpa*      CAGCTAGATTTGTCTTTAAGGCTTTGA 
*P.davidiana*        CAGCTAGATTTGTCTTTAAGGCTTTGA 
                   ***************************
