# Supplementary material for: Analysis of transcription factors among differentially expressed genes induced by drought stress in Populus davidiana
Source: 3 Biotech. 2017 Jun 30;7(3):209. doi: 10.1007/s13205-017-0858-7 (PMC5493580; doi:10.1007/s13205-017-0858-7)
Supplement: Supplementary file 5 — Supplementary material 5 (DOCX 14 kb) [file 13205_2017_858_MOESM5_ESM.docx]

**Supplementary Table S4 List of primers used for qRT-PCR analysis.**

| **Accession No.** | **Forward Sequence (5’-3’)** | **Reverse sequence (5’-3’)** |
| --- | --- | --- |
| POPTR_0013s04170 | ACCTGGAGCGACAGGACTAA | CCACTGCCTTCCTCTTCATC |
| POPTR_0016s10610 | ACCTACGAAGGGCTCCATTT | GGCTTGAAACTCGTCCTCTG |
| POPTR_0010s13400 | AAGGCTCCTCCTCTGGCTAC | TGTAGCTCCCTGTTCCCATC |
| POPTR_0014s10160 | GTCTCCTGAGCTGTCATCAG | CAGAACACCTGTGATTTTTCTCCA |
| POPTR_0014s09860 | AGCCGGTAGAGGAAGGAGAG | CTCCCCCAATCTTCTTGTGA |
| POPTR_0015s14070 | ATGCATCCCTTCGAGCTTTA | CACTTGGTGAGGCAACAGAA |
| POPTR_0016s07010 | CCCGGTTATAGTCCCAACTG | CACCATATGACGCATTGAGG |
| POPTR_0013s13340 | TTCAAGGAACCACAGCCACT | CTGGCCCACTTGTCAAAACT |
| POPTR_0017s11880 | TTGTCTCCCGGTAAACCAAG | TATCAGGCGACATGGATTGA |
| POPTR_0015s06480 | GGTGCGTGCCCTAAAGATGA | GTGTTTTTGTCAAGGAAAATCAGG |
